# Mixing Rules for Left-Handed Disordered Metamaterials: Effective-Medium and Dispersion Properties

**DOI:** 10.3390/nano14121056

**Published:** 2024-06-19

**Authors:** Ana Bărar, Stephen A. Maclean, Barry M. Gross, Doina Mănăilă-Maximean, Octavian Dănilă

**Affiliations:** 1 Electronic Technology and Reliability Department, National University of Science and Technology Politehnica Bucharest, 060082 Bucharest, Romania; ana.barar@upb.ro; 2 Chemical Engineering Department, Tandon School of Engineering, New York University, Brooklyn, New York, NY 11201, USA; 3 Optical Remote Sensing Laboratory, The City College of New York, New York, NY 10031, USA; 4 NOAA—Cooperative Science Center for Earth System Sciences and Remote Sensing Technologies, New York, NY 10031, USA; 5 Physics Department, National University of Science and Technology Politehnica Bucharest, 060082 Bucharest, Romania; 6 Academy of Romanian Scientists, 050044 Bucharest, Romania; 7 Laser Systems Department, National Institute for Physics and Nuclear Engineering, 077125 Măgurele, Ilfov, Romania

**Keywords:** mixing rules, dielectrics, composites, left-handed materials, electric permittivity, metamaterials, dispersion

## Abstract

Left-handed materials are known to exhibit exotic properties in controlling electromagnetic fields, with direct applications in negative reflection and refraction, conformal optical mapping, and electromagnetic cloaking. While typical left-handed materials are constructed periodic metal-dielectric structures, the same effect can be obtained in composite guest–host systems with no periodicity or structural order. Such systems are typically described by the effective-medium approach, in which the components of the electric permittivity tensor are determined as a function of individual material properties and doping concentration. In this paper, we extend the discussion on the mixing rules to include left-handed composite systems and highlight the exotic properties arising from the effective-medium approach in this framework in terms of effective values and dispersion properties.

## 1. Introduction

The effective-medium approach for the description of the electromagnetic response of various material composites has been used for more than a century, starting with the Maxwell–Garnett mixing rules [1,2]. The model derives an effective single value of the dielectric constant by assuming a homogeneous, isotropic dielectric host and a metallic guest comprising spherical metallic fragments having a concentration below a specific threshold. The model has since been extended to include a higher filling factor [3,4], as well as all-dielectric composites [5], metallic nanoparticles/liquid crystal two-phase systems [6,7], transient responses [8], a larger number of guest particles [9], and elliptical particles [10].

Recently, left-handed materials have gained extraordinary traction as the leading candidate for large-scale, fully integrable electromagnetic field controllers because of their ‘exotic’ properties. Such properties include hyperbolic wavefront controllers [11,12], negative reflection and refraction [13], conformal optical mapping [14], electromagnetic cloaking [15], epsilon-near-zero wave propagation [16], electromagnetically induced transparency [17] and a negative-index Kerr effect [18]. Initially, all left-handed materials were artificially created, periodically structured composites arranged in a solid structure, as this was the requirement for propagating the unit cell interaction with the electromagnetic field across the entire structure. The electromagnetic response of such structures is strongly dependent on the types of materials used (metal-dielectric or all-dielectric) and the geometry and sizes of the unit cell elements, and their relative displacement on the unit cell map. As a result, the so-called frequency-selective surfaces [19] have exhibited an on-demand control on the Fresnel coefficients (reflection, transmission, and absorption), accumulated phase [20,21], and output polarization [22]. The strong non-locality of frequency-selective surfaces [23] have also induced effects of dichroism [24] and chirality [25,26], in which two electromagnetic fields, each having certain specific properties, experience a different net effect after interacting with the frequency-selective surface. Relevant examples of the non-locality property are selective focusing [27] and chiral imaging [28].

More recently, however, the same net effect on the electromagnetic field was observed in partially ordered [29] and fully disordered structures [30,31,32], when the net effect on the electromagnetic field was resolved in terms of effective scattering properties rather than periodic unit cell effect replication. Such materials can be obtained by immersing Janus nanoparticles [33], which can exhibit a negative electric permittivity, in a positively valued permittivity host medium, such as nematic liquid crystals [34,35,36], coatings [37], and thin film structures [38]. The advantage of such structures is that they can be configured to exhibit the property of locality, as opposed to periodically ordered metasurfaces and metastructures, which are highly non-local. Moreover, obtaining disordered guest–host media with left-handed materials can be significantly more cost-effective by comparison to previously reported metamaterials. In the case of metamaterials, obtaining periodic structures with very specific conditions in terms of size and relative positioning (with near-zero error tolerance) requires a very complex technological fabrication process. Conversely, disordered media are relatively easier to fabricate, as the requirements on the size and relative positioning of metaparticles within the host media are practically nonexistent. Owing to all these properties, effective-medium approaches can be applied to left-handed materials, opening a new set of possible outcomes in terms of net effects imparted on the output field.

In this paper, we focus on the mixing rules for various effective-medium approaches to include fully disordered left-handed metamaterials as a function of the volume-filling fraction of the inclusion material and as a function of the anisotropic properties of the inclusion material. For such systems, we determine the effective dielectric permittivity and electric conductivity of the medium as functions of various conditions imposed on the constituting materials in isotropic and anisotropic cases. We also investigate the dispersion properties of such materials, highlighting the resonance properties of the symmetric and asymmetric modes. A key motivation for performing the study is the fact that, while frequency-selective surfaces require a complex, highly accurate technological fabrication process, the inclusion of left-handed materials, such as Janus metaparticles, in a liquid or liquid crystal host system is a relatively non-imposing technological process in terms of both accuracy and costs. Furthermore, the construction of a non-periodic composite also eliminates possible diffraction effects that most frequency-selective surfaces exhibit. Also, depending on the guest and/or host system, the composite structure may be addressable by external electric or magnetic fields, offering a new degree of control in all applications. For all reasons presented above, we believe this study serves to pave the way for the creation of new composite devices involving Janus metaparticles inserted into liquid crystal hosts, arranged in thin film structures, or included in metallic coatings. The generality of the study does not impose the selection of a particular frequency window. Therefore, the applications can cover all the operational electromagnetic spectrum from GHz to the visible regime.

## 2. Mixing Rules for the Electric Permittivity

To account for the most general material, the material law involving the two electric field vectors is written in tensor form:

(1) $$D=\overset{ˇ}{\epsilon }E$$

where $\overset{ˇ}{\epsilon }$ represents the absolute electric permittivity dyadic. For linear anisotropic materials, there exists a set of axes for which the matrix describing the dyadic is diagonalizable and has the following form:

(2) $$\overset{ˇ}{\epsilon }=\left[\begin{array}{ccc} \epsilon_{x} & 0 & 0\\ 0 & \epsilon_{y} & 0\\ 0 & 0 & \epsilon_{z} \end{array}\right]$$

which for long-axis ellipsoidal systems, such as nematic liquid crystals, becomes

(3) $$\overset{ˇ}{\epsilon }=\left[\begin{array}{ccc} \epsilon_{⊥} & 0 & 0\\ 0 & \epsilon_{\|} & 0\\ 0 & 0 & \epsilon_{⊥} \end{array}\right]$$

for liquid crystals below their Freedericksz transition and

(4) $$\overset{ˇ}{\epsilon }=\left[\begin{array}{ccc} \epsilon_{⊥} & 0 & 0\\ 0 & \epsilon_{⊥} & 0\\ 0 & 0 & \epsilon_{\|} \end{array}\right]$$

for liquid crystals above their Freedericksz transition [39]. The notations ‘⊥’ and ‘‖’ correspond to the directions perpendicular and parallel to the long axis of the liquid crystal molecule, respectively. For guest–host systems, the mixing rules have to account for a series of factors ranging from the independent electric permittivity values to the anisotropy of the components resulting from the molecular geometry or crystalline asymmetry. Here, we have considered the most utilized mixing rules, namely the Maxwell–Garnett and Bruggeman rules, because of their ability to complement each other in accurately describing the whole spectrum of volume-filling fractions. The Maxwell–Garnett mixing rule accurately describes the effective permittivity for low-valued volume-filling fraction (0<f<0.25), while the Bruggeman rule more accurately describes the rest of the filling fraction interval (0.25<f<1).

### 2.1. The Maxwell Garnett Mixing Rule

**Figure 1:**
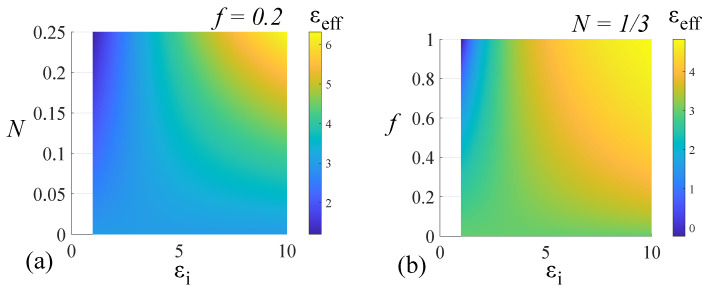
Effective permittivity of a two-component host–guest system in the Maxwell–Garnett model as a function of a positively valued permittivity inclusion particle, (**a**) inclusion filling fraction *f*, and (**b**) depolarization factor $N_{x,y,z}$.

As stated before, the most widespread mixing rule for host-guest systems is the Maxwell–Garnett rule, which typically holds for low-concentration inclusions in the host medium. Specifically, the mixing rule correctly approximates the effective value of the electric permittivity for volume-filling fractions f<0.25. In anisotropic guest–host systems, each of the permittivity components along x,y, and *z* has to be adjusted by the depolarization factors $N_{x,y,z}$. These scalar coefficients range from zero to one and are exclusively dependent on the geometry of the guest particle and degree of anisotropy of the environment [40]. For spherical inclusions, $N_{x,y,z}=1/3$; whereas for elliptic-shaped inclusions, the depolarization factor is determined via the associated elliptic integral across the geometry. Considering a host system having $\epsilon_{e}$ and an inclusion having $\epsilon_{i}$, the effective permittivity of a system in the Maxwell–Garnett model is given by

(5) $$\epsilon_{eff,x,y,z}=\epsilon_{e}+f\frac{\left(\epsilon_{i}-\epsilon_{e}\right)\epsilon_{e}}{\epsilon_{e}+\left(1-f\right)N_{x,y,z}\left(\epsilon_{i}-\epsilon_{e}\right)}$$

where *f* is the volume fraction of inclusions are in the host medium. For guest–host systems involving liquid crystal hosts, the conversion from the xyz system to the parallel and perpendicular directions is given elsewhere [39]. For our study, given that Janus metamaterials have a strong deviation from simple, symmetric geometries, we prefer a numerical appreciation to a geometry-driven value for $N_{x,y,z}$. Furthermore, we make no initial assumption on the properties of the inclusion material; rather, we perform a scan on possible values for the permittivity value of inclusions at different volume fractions. This non-assumption procedure is chosen based on two reasons: Firstly, the highly irregular geometry of the Janus metamaterial produces strong anisotropy, and given the relative rotation of individual particles with respect to each other, no apriori assumption can be made. Secondly, metamaterials are notorious for undergoing drastic changes to dielectric properties in response to any slight variations in the geometries and sizes of their component particles, and therefore, any prior assumption would not provide an accurate picture. For our investigation, we chose an isotropic host material having $\epsilon_{e}=3$. This simplification does not lead to a loss of generality in the anisotropic behavior of the system, as the directions x,y, and *z* are interchangeable because of the scanning of $N_{x,y,z}$. To serve as a benchmark, we first performed a scan of positively valued $\epsilon_{i}$ for various volume fractions and depolarizing factors. The results are presented in Figure 1.

The results presented in Figure 1a show a variation of the effective permittivity between a dielectric with a unit-value of $\epsilon_{eff}$ and a dielectric with double the value of $\epsilon_{e}$. For large *f*-values and low-valued $\epsilon_{i}$, $\epsilon_{eff}$ tends to a unit value, whereas for large *f*-values and high-valued $\epsilon_{i}$, the effective permittivity is more than double the value of $\epsilon_{e}$. For low *f*-values, we have $\epsilon_{eff}\to \epsilon_{e}$. In terms of the depolarizing factor, the effective permittivity of the system varies from an epsilon-near-zero (ENZ) material to a system with $\epsilon_{eff}>\epsilon_{e}$. The ENZ material case is supported by the fact that, for a positive $\epsilon_{i}$, the numerator of the mixing law expression tends to zero for $\epsilon_{i}=\epsilon_{e}$, while the denominator remains finite and constant for finite values of $\epsilon_{i}$. A special case is the spherical geometry nanoparticle inclusion, for which $N_{x,y,z}=1/3$. For this case, $\epsilon_{eff}$ ranges from 1.8 to 4.2 as a function of the inclusion permittivity $\epsilon_{i}$.

When considering negatively valued $\epsilon_{i}$ materials with the appropriately chosen values of *f* and $N_{x,y,z}$, the denominator of the mixing rule equation tends to zero. The relation between the component properties in order to obtain this regime is

(6) $$|\epsilon_{i}|=\epsilon_{e}\frac{1-\left(1-f\right)N}{\left(1-f\right)N}$$

while the numerator remains finite for all finite values of $\epsilon_{i}$. It is, therefore, possible to achieve extremely large values for $\epsilon_{eff}$ by providing an appropriate filling fraction to accommodate the preset parameters $\epsilon_{i}$ and $N_{x,y,z}$. Regardless of the type of material used for the inclusion, a method of accurately measuring the depolarization factor $N_{x,y,z}$ of arbitrary-shaped metaparticles can be devised in the regions of $\epsilon_{eff}$ offering low sensitivity to both *f* and $N_{x,y,z}$. Thus, the depolarization factor is achieved from the mixing rule as:

(7) $$N_{x,y,z}\left(f,\epsilon_{eff,x,y,z}\right)=\frac{f\epsilon_{e}}{\left(1-f\right)\left(\epsilon_{eff,x,y,z}-\epsilon_{e}\right)}-\frac{\epsilon_{e}}{\left(1-f\right)\left(\epsilon_{i}-\epsilon_{e}\right)}$$

The condition of low sensitivity dictates that both $\epsilon_{eff,x,y,z}$ and $N_{x,y,z}$ are functions of *f*, which can be evaluated with a certain standard deviation. The low-sensitivity condition ensures that the standard deviation of the volume fraction and, indirectly, of the measured effective-medium value introduces minimum error in the determination of the value of $N_{x,y,z}$. The low-sensitivity region can be evaluated by setting extremum conditions on the derivatives, giving the derivative of $\epsilon_{eff,x,y,z}$ expression as:

(8) $$\frac{\partial N}{\partial \epsilon_{eff,x,y,z}}=-\frac{f}{1-f}\frac{\epsilon_{e}}{{\left(\epsilon_{eff,x,y,z}-\epsilon_{e}\right)}^{2}}$$

and the low-sensitivity region is located around

(9) $$\epsilon_{eff,x,y,z}=2f\left(\epsilon_{e}\pm \sqrt{\left(1-f\right)\left(\epsilon_{e}+1\right)\epsilon_{e}}\right)$$

**Figure 2:**
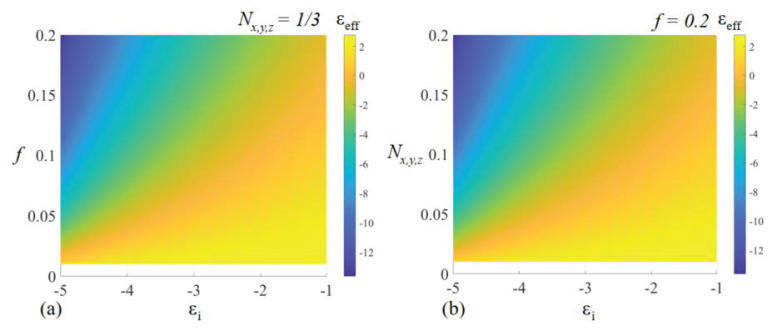
Effective permittivity of a two-component host–guest system in the Maxwell–Garnett model as a function of a negatively valued inclusion metaparticle, (**a**) the inclusion volume-filling fraction *f*, and (**b**) depolarization factor $N_{x,y,z}$.

Similarly, the derivative in *f* is

(10) $$\frac{\partial N}{\partial f}=\frac{\epsilon_{e}}{{\left(1-f\right)}^{2}}\left(\frac{1}{\epsilon_{eff,x,y,z}-\epsilon_{e}}-\frac{1}{\epsilon_{i}-\epsilon_{e}}\right)$$

having a corresponding low-sensitivity region for $\epsilon_{eff,x,y,z}≃\epsilon_{i}$. The low-sensitivity regions conditions can, however, be bypassed when a high-accuracy measurement can be performed on both $\epsilon_{eff,x,y,z}$ and the volume fraction *f* of the metaparticle inclusion. Based on all the above considerations, Figure 2 depicts the values of the effective permittivity as a function of the negatively valued $\epsilon_{i}$ of the metaparticle inclusion and of either the volume-filling fraction *f* or the depolarization factor $N_{x,y,z}$, both taken in a region where the mixing rule exhibits low sensitivity.

### 2.2. The Bruggeman Mixing Rule

One of the most widely accepted extensions of the Maxwell–Garnett mixing rule is the Bruggeman rule, which offers a very good approximation of the Maxwell–Garnett mixing rule at high *f*-values. Since it considers large values of the volume fraction, the guest–host system can be viewed symmetrically if the two media are isotropic. The rule is a direct result of Gauss’s Law for electric fields. Considering the guest–host system lacking in net charges, the net electric flux through an arbitrary closed surface $\phi_{e}$ in the volume of the system is zero. When averaging across all scattering particles inside the closed surface, we obtain

(11) $$\oint \epsilon_{n}\left(r\right)E_{n}\left(r\right)dA=\epsilon_{eff}\oint E_{⊥}dA$$

where $\epsilon_{n}\left(r\right)$ is the component permittivity, $E_{n}\left(r\right)$ is the normal component of the scattered field of each microscopic component, $E_{⊥}$ is the normal component of the macroscopic field, and dA is the closed surface element taken in the volume of the guest–host system. Since the surface is chosen arbitrarily, it can be made sufficiently small to enclose a single inclusion $\epsilon_{i}$ in the host medium $\epsilon_{e}$. Following this assumption, the Clausius–Mossoti relation can be extended to the Bruggeman mixing rule as

(12) $$1-\sum_{j}f_{j}\frac{\epsilon_{j}-\epsilon_{eff}}{\epsilon_{j}+2\epsilon_{eff}}=0$$

where *j* takes into account all the participants in the mixture (host and j−1 guests if viewed asymmetrically). For a two-component system, the effective permittivity is

(13) $$\epsilon_{eff}=\frac{1}{4}\left(Q+\sqrt{Q^{2}+8\epsilon_{e}\epsilon_{i}}\right)$$

where

(14) $$Q=\left(3f-1\right)\epsilon_{i}+\left(3\left(1-f\right)-1\right)\epsilon_{e}$$

**Figure 3:**
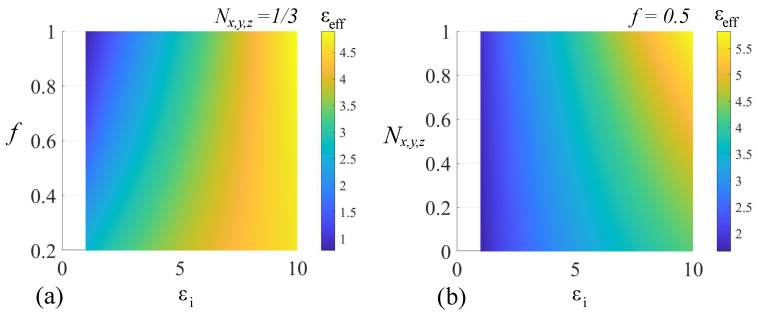
The adapted Bruggeman mixing rule for positively valued permittivities in the case of (**a**) fixed depolarization factor and (**b**) a fixed-volume-filling fraction.

In its current form, the mixing rule only only accounts for isotropic media, leaving out anisotropy and element-shape-induced effects, such as the depolarization factor. Here, we propose the extension of the mixing rule to encompass anisotropic inclusions with arbitrary shapes by rewriting *Q* as

(15) $$Q^{\prime }=\left(3f-1\right)N_{x,y,z}\epsilon_{i}+\left(3\left(1-f\right)-1\right)\epsilon_{e}$$

while the rest of the law remains unchanged. For validation, we consider an $\epsilon_{e}=3$ host medium that contains an inclusion $\epsilon_{i}$ with variable volume fraction *f* and depolarization factor $N_{x,y,z}$ to account for an arbitrary shape. The results for a positively valued inclusion permittivity $\epsilon_{i}$ are presented in Figure 3.

In the case of spherical inclusions ($N_{x,y,z}=1/3$) with positive $\epsilon_{i}$, the effective permittivity values are solely positive. For f=1, we have $\epsilon_{eff}=\epsilon_{i}$, as expected. In the case of, we have $\epsilon_{eff}=\epsilon_{e}$. The limits are respected for a fixed-volume fraction f=0.5 and variable $N_{x,y,z}$. Also, for f=1/3, it follows that $Q^{\prime }=\epsilon_{e}$, and the effective permittivity is

(16) $$\epsilon_{eff}=\frac{1}{4}\left(\epsilon_{e}+\sqrt{\epsilon_{e}^{2}+8\epsilon_{e}\epsilon_{i}}\right)$$

Similarly, for f=2/3, we obtain $Q^{\prime }=\epsilon_{i}$, the effective permittivity $\epsilon_{eff}=N_{x,y,z}\epsilon_{i}$, and the effective permittivity expression:

(17) $$\epsilon_{eff}=\frac{1}{4}\left(\epsilon_{i}N_{x,y,z}+\sqrt{\epsilon_{i}^{2}N_{x,y,z}^{2}+8\epsilon_{e}\epsilon_{i}}\right)$$

When considering a negatively valued $\epsilon_{i}$, a real-valued $\epsilon_{eff}$ is obtained only for the following condition:

(18) $$\Gamma =Q^{2}+8\epsilon_{i}\epsilon_{e}>0$$

Inserting

(19) $$\begin{array}{cc} \alpha =3f-1; & \beta =3\left(1-f\right)-1=2-3f, \end{array}$$

and solving for $\epsilon_{i}$, the zeroes for the equation are

(20) $$\Gamma_{1}\left(f,N_{x,y,z}\right)=-\frac{\sqrt{\alpha \beta N_{x,y,z}+4}}{\alpha^{2}N_{x,y,z}^{2}}\left(2+\sqrt{\alpha \beta N_{x,y,z}+4}\right)\epsilon_{e}$$

(21) $$\Gamma_{2}\left(f,N_{x,y,z}\right)=-\frac{\sqrt{\alpha \beta N_{x,y,z}+4}}{\alpha^{2}N_{x,y,z}^{2}}\left(\sqrt{\alpha \beta N_{x,y,z}+4}-2\right)\epsilon_{e}$$

**Figure 4:**
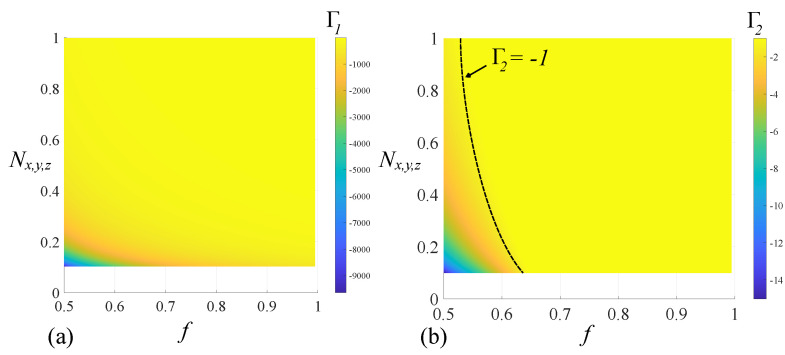
Root values (**a**) $\Gamma_{1}$ and (**b**) $\Gamma_{2}$ as a functions of *f* and $N_{x,y,z}$ associated with left−handed materials that support propagating waves. For $\Gamma_{2}$, the dotted line corresponds to the threshold value $\epsilon_{thr}=-1$.

For $\epsilon_{i}\in \left(-\infty ,\Gamma_{1}\right)\cup \left(\Gamma_{2},-1\right)$, real values of $\epsilon_{eff}$ are obtained. Conversely, $\epsilon_{i}\in \left(\Gamma_{1},\Gamma_{2}\right)$, gives a complex-valued $\epsilon_{eff}$, corresponding to an extinction of the radiation and an evanescent radiating regime. The values of $\Gamma_{1}$ and $\Gamma_{2}$ as functions of *f* and $N_{x,y,z}$ are presented in Figure 4a,b, respectively. For all possible combinations of *f* and $N_{x,y,z}$, $\Gamma_{1}$ is negative and has a relatively large value with respect to the $\epsilon_{i,thr}=-1$ threshold. This implies that any $\epsilon_{i}<\Gamma_{1}$ can produce a composite that can sustain a propagating wave. However, for *f*-values close to the unit value, $\Gamma_{1}<-100$, which makes the condition $\epsilon_{i}<\Gamma_{1}$ almost impossible to satisfy experimentally. As such, the other region of interest is, therefore, $\epsilon_{i}\in \left(\Gamma_{2},-1\right)$. However, $\Gamma_{2}$ can have positive values for

(22) $$\sqrt{\alpha \beta N_{x,y,z}+4}-2<0$$

which corresponds to αβ<0. Therefore, we need to determine the values of the volume fraction *f* that lead to a negative $\Gamma_{2}$, i.e., a value lower than the threshold: we rewrite the condition $\Gamma_{2}<\epsilon_{i,thr}=-1$, and after some calculation, we obtain the inequality

(23) $${\left(\alpha \beta N_{x,y,z}+4\right)}^{2}-2\left(\alpha \beta N_{x,y,z}+4\right)\left(\frac{\alpha^{2}N_{x,y,z}^{2}}{\epsilon_{e}}-1\right)+\frac{\alpha^{4}N_{x,y,z}^{4}}{\epsilon_{e}^{2}}<0$$

The above equation can be solved numerically by setting fixed values of *f* and $N_{x,y,z}$ and solving $\Gamma_{2}=\epsilon_{i,thr}$. We, therefore, impose $f\in \left(0.5,1\right)$ and $N_{x,y,z}\in \left(0.1,1\right)$. Under these conditions, the geometric locus corresponding to the solution of the above equation is the dotted line depicted in Figure 4b.

## 3. Mixing Rules for the Electric Conductivity

**Figure 5:**
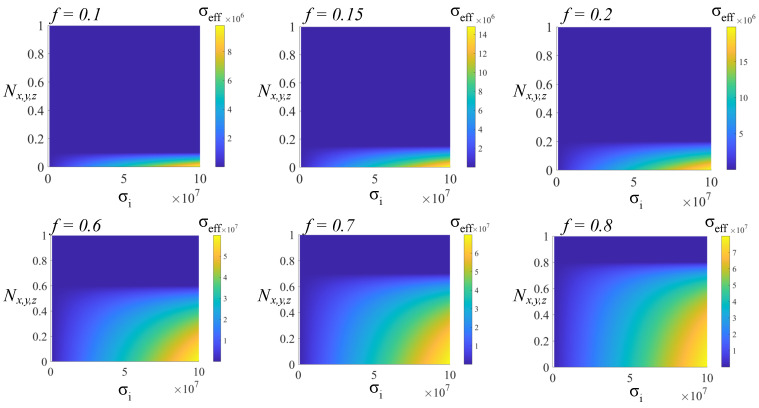
The effective conductivity of a two-phase symmetric system as a function of the depolarization factor $N_{x,y,z}$ and the conductivity $\sigma_{i}$ of a metallic inclusion for fixed values of volume-filling fractions *f*. The conductivity of the host medium is $\sigma_{e}=10^{-5}$ S/m.

The characterization of real composite media has to take into account the electric conductivity σ, which describes the parasitic effect of energy leakage through electric currents and, indirectly, its outward dissipation through the Joule effect. Regardless of the type of material (left-handed or right-handed), the electric conductivity is a positively valued scalar or dyadic that depends on the anisotropy of the components. Therefore, a left-handed system behaves in the same manner as a right-handed system from an energy-loss point of view. The mixing rule for the electric conductivity is expressed in its most general form when viewed as a property of a symmetric guest–host system. Using the Bruggeman interpretation on a two-phase system, characterized by a volume fraction *f* and a depolarization factor $N_{x,y,z}$, the effective electric conductivity for randomly oriented ellipsoids is [41]:

(24) $$\frac{f\left(\sigma_{i}-\sigma_{eff}\right)}{\sigma_{i}+\sigma_{eff}\left(1-N_{x,y,z}\right)/N_{x,y,z}}+\frac{\left(1-f\right)\left(\sigma_{e}-\sigma_{eff}\right)}{\sigma_{e}+\sigma_{eff}\left(1-N_{x,y,z}\right)/N_{x,y,z}}=0$$

where $\sigma_{i}$ and $\sigma_{e}$ represent the electrical conductivities of the two phases, asymmetrically viewed as inclusion and host, respectively. Solving for $\epsilon_{eff}$, the above relation becomes

(25) $$\alpha \sigma_{eff}^{2}+\left(\left(\alpha \sigma_{e}-\sigma_{i}\right)-f\left(\alpha +1\right)\left(\sigma_{e}-\sigma_{i}\right)\right)\sigma_{eff}+\sigma_{i}\sigma_{e}=0$$

where $\alpha =\frac{1-N_{x,y,z}}{N_{x,y,z}}$. Figure 5 presents the effective conductivity of a host dielectric system with $\sigma_{e}=10^{-5}$ S/m and metallic inclusions having $\sigma_{i}\in \left(10^{7},10^{8}\right)$ S/m for low- and high-level inclusion fractions *f*.

## 4. Dispersion Properties

### Zero-Order Dispersion

**Figure 6:**
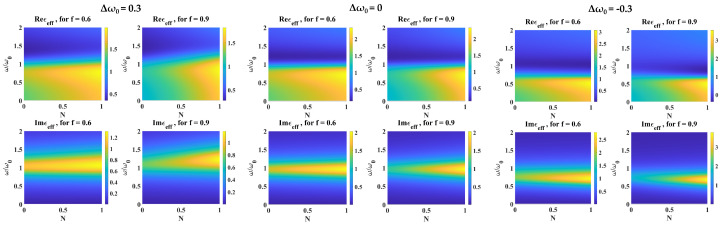
Effective permittivity $\epsilon_{eff}$ of a two−phase system as a function of normalized frequency $\omega /\omega_{0}$ and depolarization factor *N* for fixed values of volume-filling fractions *f* at resonance frequency gaps $\Delta \omega_{0}=0,\pm 0.3\omega_{0}$.

For an applied AC signal, the two-phase material under study exhibits dispersion, in which both the electric permittivity and electric conductivity become frequency-dependent. Most notably, the Drude–Lorentz model establishes a sign invariance of the $\epsilon \left(\omega \right)$, because the minus sign of permittivity only signifies an accumulated phase of π between the driving force and the response of the material. In the zero-order dispersion approximation, in which a pure sinusoidal field is acting upon the system, the complex relative permittivity of each material is given by the Drude–Lorentz oscillator model:

(26) $$\begin{array}{c} \epsilon \left(\omega \right)=1+\sum_{j}\frac{\omega_{p}^{2}}{\omega_{0j}^{2}-\omega^{2}-i\omega \gamma } \end{array}$$

where $\omega_{p}$ is the plasma frequency, $\omega_{0j}$ is the *j*-th resonance frequency, and γ is the attenuation factor, which is related to the relaxation time τ as γ=1/τ. In the non-interacting picture, both host and inclusion materials have independent dispersion properties and, therefore, behave as uncoupled oscillators. This case is usually obtained for dilute dielectric mixtures, in which the interaction forces can be neglected. For materials exhibiting resonances that are far apart, the overall behavior is dominated by the individual resonance behavior of each material, as it is known. When resonances are sufficiently close, however, the overall response becomes strongly dependent on the gap in the two resonances Δω, with added deviations introduced by the filling factor $N_{x,y,z}$ and filling fraction *f*. For our study, we considered the more interesting case of small-gap resonance frequencies. We used Equations (17) and (26), in which we only considered one resonance frequency per material phase. To clearly exhibit the behavior, we normalized both plasma frequencies $\omega_{p}$ to reduce scaling factors in the mixing law. Also, the effective frequency response was referenced to the resonance frequency of the host material $\omega_{0}$, and the normalized frequency $\omega /omega_{0}$ was scanned from $0.01\omega_{0}$ to $100\omega_{0}$. The relaxation phenomena of both dielectrics were modeled by individual attenuation factors (i.e., $\gamma_{e}=0.5\omega_{0}$ for the host medium and $\gamma_{i}=0.2$ for the inclusion). Because of the symmetry of the Bruggeman mixing law, the component values were interchangeable, with the notable distinction that any asymmetry is induced by the inclusion, and modeled by the depolarization factor. Figure 6 presents the simulated results of the effective response $\epsilon_{eff}\left(\omega \right)$ for a two-phase dielectric mixture with various fixed-value resonance frequency gaps $\Delta \omega_{0}=\omega_{0i}-\omega_{0e}$.

The $\Delta \omega_{0}=0$ case serves as a validation of the previously known frequency response. The composite mixture response exhibits a resonance at $\omega_{0}$ as expected, and the attenuation factor of the composite is modulated by both material properties. For $\Delta \omega_{0}=0.3\omega_{0}$, the resonance of the effective response is shifted to $\omega_{0}^{\left(+\right)}=1.12\omega_{0}$. In contrast, for $\Delta \omega_{0}=-0.3\omega_{0}$, the resonance is shifted to $\omega_{0}^{\left(-\right)}=0.7\omega_{0}$. The asymmetry between resonance shifts can be attributed to the different relaxation times of the individual phases, leading to different attenuation factors. The absorption spectra of the mixture, expressed by the imaginary part of the effective permittivity, have a symmetric bell shape in the case of $\Delta \omega_{0}=0$ and become asymmetrically skewed as the resonance gap either increases or decreases.

## 5. Conclusions

In this paper, we conducted a study of the effective-medium properties of two-phase guest–host systems, by focusing on the changes induced by the inclusion of a left-handed material in a typical host system. The systems were treated both asymmetrically, via the Maxwell–Garnett mixing rule, and symmetrically, via the Bruggeman mixing rule. To account for anisotropic effects, we included the depolarization factor along each axis $N_{x,y,z}$ and scanned for all possible values. As opposed to typical materials in which $\epsilon_{i}$ and $\epsilon_{e}$ are positive, the mathematical form of the mixing laws enables a continuous spectrum for $\epsilon_{eff}$, in which the values of $\epsilon_{eff}$ are comparable with the guest and host permittivities. For left-handed materials, however, the mixing laws change significantly, leading to enormous permittivity values or to evanescent wave media because of complex-valued $\epsilon_{eff}$. A similar effect was observed for conductivity, in which a real-valued $\sigma_{eff}$ was obtained only for certain intervals determined by the depolarization factor. Finally, zero-order dispersion in the Drude–Lorentz model was analyzed for a symmetric two-phase system using the Bruggeman mixing law. While the left-handed material induced no variation in the dispersion properties, the depolarization factor was directly responsible for the frequency response of $\epsilon_{eff}$. These derived properties, thus highlighted, are a starting point for many applications in the engineering of disordered materials that exhibit designer electromagnetic properties, such as disordered metamaterials.

## Data Availability

Data are contained within the article.

## References

[1] Maxwell Garnett J.C. (1905). Colours in metallic glasses, metallic films, and in metallic solution. Philos. Trans. A.

[2] Markel V.A. (2016). Introduction to the maxwell garnett approximation: Tutorial. J. Opt. Soc. Am. A.

[3] Bruggeman D.A.G. (1935). Berechnung verschiedener physikalischer konstanten von heterogenen substanzen. i. dielektrizitätskonstanten und leitfähigkeiten der mischkörper aus isotropen substanzen. Ann. Phys..

[4] Polder D., van Santen J.H. (1946). The effective permeability of mixtures of solids. Physica.

[5] Wu Y., Zhao X., Li F., Fan Z. (2003). Evaluation of mixing rules for dielectric constants of composite dielectrics by mc-fem calculation on 3d cubic lattice. J. Electroceramics.

[6] Manaila-Maximean D. (2021). Effective permittivity of a multi-phase system: Nanoparticle-doped polymer-dispersed liquid crystal films. Molecules.

[7] Ganea C.P., Cîrcu V., Manaila-Maximean D. (2020). Effect of titanium oxide nanoparticles on the dielectric properties and ionic conductivity of a new smectic bis-imidazolium salt with dodecyl sulfate anion and cyanobiphenyl mesogenic groups. J. Mol. Liq..

[8] Kristensson G., Rikte S., Sihvola A. (1998). Mixing formulas in time domain. J. Opt. Soc. Am. A.

[9] Wu K., Li J., von Salzen K., Zhang F. (2018). Explicit solutions to the mixing rules with three-component inclusions. J. Quant. Spectrosc. Radiat. Transf..

[10] Sihvola A. (2000). Mixing rules with complex dielectric coefficients. Subsurf. Sens. Technol. Appl..

[11] Gomez-Diaz J.S., Tymchenko M., Alù A. (2015). Hyperbolic plasmons and topological transitions over uniaxial metasurfaces. Phys. Rev. Lett..

[12] Hu G., Krasnok A., Mazor Y., Qiu C., Alù A. (2020). Moiré hyperbolic metasurfaces. Nano Lett..

[13] Yu N., Capasso F. (2014). Flat optics with designer metasurfaces. Nat. Mater..

[14] Leonhardt U. (2006). Optical conformal mapping. Science.

[15] Schurig D., Mock J.J., Justice B.J., Cummer S.A., Pendry J.B., Starr A.F., Smith D.R. (2006). Metamaterial electromagnetic cloak at microwave frequencies. Science.

[16] Alù A., Silveirinha M.G., Salandrino A., Engheta N. (2007). Epsilon-near-zero metamaterials and electromagnetic sources: Tailoring the radiation phase pattern. Phys. Rev. B.

[17] Wang B.-X., Duan G., Lv W., Tao Y., Xiong H., Zhang D.-Q., Yang G., Shu F.-Z. (2023). Design and experimental realization of triple-band electromagnetically induced transparency terahertz metamaterials employing two big-bright modes for sensing applications. Nanoscale.

[18] Nookala N., Lee J., Tymchenko M., Gomez-Diaz J.S., Demmerle F., Boehm G., Lai K., Shvets G., Amann M., Alu A. (2016). Ultrathin gradient nonlinear metasurface with a giant nonlinear response. Optica.

[19] Munk B.A. (2000). Frequency Selective Surfaces: Theory and Design.

[20] Neshev D., Aharonovich I. (2018). Optical metasurfaces: New generation building blocks for multi-functional optics. Light Sci. Appl..

[21] Engleberg J., Levy U. (1991). The advantages of metalenses over diffractive lenses. Nat. Commun..

[22] Papakostas A., Potts A., Bagnall D.M., Prosvirnin S.L., Coles H.J., Zheludev N.I. (2003). Optical manifestations of planar chirality. Phys. Rev. Lett..

[23] Overvig A., Alù A. (2022). Diffractive nonlocal metasurfaces. Laser Photon. Rev..

[24] Wang S., Deng Z.L., Wang Y., Zhou Q., Wang X., Cao Y., Guan B.O., Xiao S., Li X. (2021). Arbitrart polarization conversion dichroism metasurfaces for all-in-one full poincaré sphere polarizers. Light Sci. Appl..

[25] Tanaka K., Arslan D., Fasold S., Steinert M., Sautter J., Falkner M., Pertsch T., Decker M., Staude I. (2020). Chiral bilayer all-dielectric metasurfaces. ACS Nano.

[26] Kim Y., Kim H., Yang Y., Badloe T., Jeon N., Rho J. (2022). Three-dimensional artificial chirality towards low-cost and ultra-sensitive enantioselective sensing. Nanoscale.

[27] Wang S., Wang X., Kan Q., Ye J., Feng S., Sun W., Han P., Qu S., Zhang Y. (2015). Spin-selected focusing and imaging based on metasurface lens. Opt. Express.

[28] Basiri A., Chen X., Bai J., Amrollahi P., Carpenter J., Holman Z., Wang C., Yao Y. (2019). Nature-inspired chiral metasurfaces for circular polarization detection and full-stokes polarimetric measurements. Light Sci. Appl..

[29] Chen K., Ding G., Hu G., Jin Z., Zhao J., Feng Y., Jiang T., Alù A., Qiu C.W. (2019). Directional janus metasurface. Adv. Mater..

[30] Yu S., Qiu C.W., Chong Y., Torquato S., Park N. (2021). Engineered disorder in photonics. Nat. Rev..

[31] Hu Z., Liu C., Li G. (2023). Disordered optical metasurfaces: From light manipulation to energy harvesting. Adv. Phys. X.

[32] Zaiser M., Zapperi S. (2023). Disordered mechanical metamaterials. Nat. Rev. Phys..

[33] Landon P.B., Mo A.H., Printz A.D., Emerson C., Zhang C., Janetanakit W., Colburn D.A., Akkiraju S., Dossou S., Chong B. (2015). Asymmetric colloidal janus particle formation is core-size-dependent. Langmuir.

[34] Conradi M., Ravnik M., Bele M., Zorko M., Zumer S., Musevic I. (2009). Janus nematic colloids. Soft Matter.

[35] Sahu D.K., Dhara S. (2020). Measuring electric-field-induced dipole moments of metal-dielectric janus particles in a nematic liquid crystal. Phys. Rev. Appl..

[36] Braun L.B., Zentel R. (2019). Functional liquid crystalline particles and beyond. Liq. Cryst..

[37] Yasuda H., Matsuno R., Koito N., Hosoda H., Tani T., Naya M. (2017). Anti-reflective coating for visible light using a silver nanodisc metasurface with a refractive index of less than 1.0. Appl. Phys. Lett..

[38] Yu P., Li J., Zhang S., Jin Z., Schütz G., Qiu C.-W. (2018). Dynamic janus metasurfaces in the visible spectral region. Nano Lett..

[39] Fuh A.Y.G., Lee W., Huang Y.C. (2013). Derivation of extended maxwell garnett formula for carbon-nanotube-doped nematic liquid crystal. Liq. Cryst..

[40] Sihvola A. (2005). Metamaterials and depolarization factors. Prog. Electromagn. Res..

[41] McLachalan D.S., Blaszkiewicz M., Newnham R.E. (1990). Electrical resistivity of composites. J. Am. Cream. Soc..

